# Epigallocatechin gallate modulates ferroptosis through downregulation of tsRNA-13502 in non-small cell lung cancer

**DOI:** 10.1186/s12935-024-03391-5

**Published:** 2024-06-05

**Authors:** Shun Wang, Ruohuang Wang, Dingtao Hu, Caoxu Zhang, Peng Cao, Jie Huang, Baoqing Wang

**Affiliations:** 1 Department of Respiratory Medicine, Shanghai Xuhui Central Hospital, Zhongshan-Xuhui Hospital, Fudan University, Shanghai, 200031 China; 2 Department of Otolaryngology, The Second Affiliated Hospital of the Naval Military Medical University (Shanghai Changzheng Hospital), Shanghai, 200003 China; 3 Clinical Cancer Institute, Center for Translational Medicine, Naval Medical University, Shanghai, 200433 China; 4 State Key Laboratory of Medical Genomics, Department of Molecular Diagnostics, Department of Endocrinology, The Core Laboratory in Medical Center of Clinical Research, Shanghai Ninth People’s Hospital, Shanghai Jiaotong University School of Medicine, Shanghai, 200011 China; 5 Department of Interventional Pulmonology，Anhui Chest Hospital, Hefei, 230022 China; 6 Department of Respiratory Medicine, Zhongshan Hospital, Fudan University, Shanghai, 200031 China

**Keywords:** Epigallocatechin gallate (EGCG), Ferroptosis, tsRNA, NSCLC

## Abstract

Ferroptosis, an iron-dependent regulated cell death mechanism, holds significant promise as a therapeutic strategy in oncology. In the current study, we explored the regulatory effects of epigallocatechin gallate (EGCG), a prominent polyphenol in green tea, on ferroptosis and its potential therapeutic implications for non-small cell lung cancer (NSCLC). Treatment of NSCLC cell lines with varying concentrations of EGCG resulted in a notable suppression of cell proliferation, as evidenced by a reduction in Ki67 immunofluorescence staining. Western blot analyses demonstrated that EGCG treatment led to a decrease in the expression of glutathione peroxidase 4 (GPX4) and solute carrier family 7 member 11 (SLC7A11) while increasing the levels of acyl-CoA synthetase long-chain family member 4 (ACSL4). These molecular changes were accompanied by an increase in intracellular iron, malondialdehyde (MDA), and reactive oxygen species (ROS), alongside ultrastructural alterations characteristic of ferroptosis. Through small RNA sequencing and RT-qPCR, transfer RNA-derived small RNA 13502 (tsRNA-13502) was identified as a significant target of EGCG action, with its expression being upregulated in NSCLC tissues compared to adjacent non-tumorous tissues. EGCG was found to modulate the ferroptosis pathway by downregulating tsRNA-13502 and altering the expression of key ferroptosis regulators (GPX4/SLC7A11 and ACSL4), thereby promoting the accumulation of iron, MDA, and ROS, and ultimately inducing ferroptosis in NSCLC cells. This study elucidates EGCG’s multifaceted mechanisms of action, underscoring the modulation of ferroptosis as a viable therapeutic approach for enhancing NSCLC treatment outcomes.

**Supplementary Information:**

The online version contains supplementary material available at 10.1186/s12935-024-03391-5.

## Introduction

Lung cancer remains the principal cause of tumor-associated mortality globally, with non-small cell lung cancer (NSCLC) constituting approximately 85% of all cases. The current therapeutic strategies yield a disconcertingly low 5-year survival rate of less than 15% [1, 2]. Standard treatment modalities for NSCLC encompass surgical resection, radiation therapy, chemotherapy, targeted therapy, and immunotherapy [3]. Although the primary goal of these treatments is curative, the late-stage diagnosis, coupled with the recurrence and resistance of the disease, presents significant obstacles to optimal NSCLC management [4, 5]. Consequently, the imperative to identify novel and efficacious treatment strategies for NSCLC is ever-pressing.

Epigallocatechin-3-gallate (EGCG), a major polyphenolic compound prevalent in green tea, demonstrates extensive bioactivity [6]. Its multifaceted biological effects, including anti-proliferative, anti-inflammatory, and anti-mutagenic properties, render it a subject of rigorous investigation as a potential cancer chemopreventive agent [7]. EGCG derivatives hinder the viability of NSCLC cells, induce cell cycle disruption, and trigger apoptotic cascades. These effects are further potentiated in combination with cisplatin, resulting in the termination of neoplastic proliferation [8, 9]. EGCG derivatives impede the viability of NSCLC cells, cause cell cycle perturbations, and induce apoptotic cascades. These effects are further enhanced in synergy with cisplatin, culminating in the abrogation of neoplastic expansion [10]. EGCG effectively curbs the self-renewal capacity of stem-like lung cancer cells through its selective interaction with CLOCK or RXRα [11, 12]. The cooperative effect of metformin and EGCG-activated Nrf2/HO-1 signaling pathway, facilitated by SIRT1-mediated Nrf2 deacetylation, enhances the susceptibility of NSCLC to EGCG modulation by promoting reactive oxygen species (ROS) generation and apoptosis [13]. However, the regulatory dynamics and molecular mechanisms underpinning EGCG’s influence on NSCLC remain elusive and warrant comprehensive elucidation.

Ferroptosis, a form of programmed cell death primarily instigated by iron-dependent lipid peroxidation, has recently gained recognition [14]. Its unique attributes, which diverge significantly from traditional cell death pathways such as apoptosis in both mechanistic and morphological aspects, have garnered substantial attention within the realm of cancer research in recent years [15]. This surge of interest is attributable, in part, to its potential as an innovative pathway to enhance cancer therapy. Extant literature delineates the complex regulatory functions of several signaling pathways, encompassing MT1DP/miR-365a-3p/NRF2 [16], NRF2 [17], and Nrf2/ HO-1 [18], in modulating the vulnerability of lung cancer cells to ferroptosis. Despite preliminary research indicating the potential for ROS induction by EGCG, the definitive role of EGCG in inhibiting cancer cell proliferation via ferroptosis promotion remains an area necessitating thorough elucidation.

tRNA-derived small RNAs (tsRNAs), a recently identified subset of noncoding small RNAs, are partitioned into stress-induced tRNA-derived RNAs (tiRNAs) and tRNA-derived fragments (tRFs). These tsRNAs are implicated in a variety of biological processes, demonstrating their pivotal roles [19, 20]. They exert regulatory influence over gene silencing, RNA stability, reverse transcription, and translation, processes intimately linked with cellular proliferation, migration, cell cycle progression, and apoptosis [19]. An expanding corpus of research underscores the critical involvement of tsRNAs in the pathogenesis of lung cancer. Wang et al. presented an in-depth exploration of tRF and tiRNA expression profiles in lung adenocarcinoma (LUAD), identifying three previously unreported downregulated tRF-1s potentially implicated in the evolution and progression of LUAD through nervous system development and the MAPK signaling pathway [21]. AS-tDR-007333 was reported to enhance NSCLC malignancy via the HSPB1/MED29 and ELK4/MED29 axes [22]. Furthermore, a stem-loop RT-qPCR analysis of serum samples from 96 NSCLC patients, 96 healthy controls, and pre-and post-operative serum samples from 20 NSCLC patients revealed the potential diagnostic significance of tRF-31-79MP9P9NH57SD in NSCLC [23]. Hu et al. discovered that tsRNA-5001a promoted LUAD cell proliferation and was correlated with postoperative recurrence in LUAD patients [24]. Nevertheless, considerable gaps persist in our understanding of tsRNAs’ roles in non-small cell lung cancer.

In this study, we probe the role of EGCG by sequencing small RNAs in NSCLC and exploring potential underlying mechanisms. We determined that tsRNA-13502 was upregulated in NSCLC and that EGCG could induce ferroptosis by downregulating tsRNA-13502. Our findings corroborate the anticancer potential of EGCG in NSCLC through ferroptosis and suggest that tsRNA-13502 may represent a viable therapeutic target for NSCLC.

## Method and materials

### Cell culture

A549 and H1299 cells were purchased from iCell Bioscience Inc. (Shanghai, China). Cells were maintained at 37 °C in 5% CO_2_ humidified air in RPMI 1640 medium containing 10% FBS (S10099-141, GIBCO).

### CCK8 assay

The CCK-8 assay was performed to evaluate cell growth. Briefly, A549 and H1299 cells were cultured at a density of 2000 cells per well. Cells were treated with different concentrations of EGCG (0, 20, 40, 60, 80, and 100 µM). CCK-8 reagent was added at the indicated time points (0 h and 72 h) and incubated for 2 h at 37 °C. The absorbance was tested at 450 nm. Based on these results, we have chosen values near ½ IC50 as the three working EGCG concentrations.

### Immunofluorescence staining

A549 and H1299 cells were fixed with 4% paraformaldehyde, permeabilized with 0.5% Triton X-100, and blocked with 3% BSA. After incubation of the primary antibody at 4 °C overnight and treatment with the secondary antibody (Alexa Fluor 488), DAPI staining was performed. Fluorescence was observed under the microscope (20× and 40× magnification).

### Western blot analysis

Total protein was isolated using radioimmunoprecipitation and lysis buffer. The extracted proteins were treated with 10% SDS-PAGE and transferred to polyvinylidene fluoride membranes. Membranes were blocked with 5% milk and incubated with the primary antibodies overnight at 4 °C, followed by incubation with the appropriate horseradish peroxidase-conjugated secondary antibodies. Signals were detected using an Enhanced Chemiluminescence Detection Kit (Cell Signaling Technology, Danvers, MA, USA). Antibodies against GPX4 (1: 5000, ab125066, Abcam), SLC7A11 (1:1000, 26864-1-AP, Abcam), ACSL4 (1:50,000, ab155282, Abcam), and GAPDH (1:15,000, 60004-I-Ig, Proteintech) were used as primary antibodies.

### Detection of malondialdehyde (MDA), ferrous iron (Fe^2+^), reactive oxygen species (ROS), and lipid ROS

MDA content was determined using an assay kit from Beyotime, Fe^2+^ was determined using an assay kit from Applygen according to the manufacturer’s protocol, and the kit from Solarbio determined ROS, lipid ROS was determined using a BODIPY 581/591 C11 from MCE by Flow CytoMetry.

### Transmission electron microscopy (TEM) observation of ferroptosis

A549 and H1299 cells were first fixed with formaldehyde, rinsed in phosphate buffer, and then stabilized with osmium tetroxide. Dehydration was achieved by a series of ethanol concentrations followed by three immersions in 100% ethanol. After dehydration, the samples were infiltrated with epoxy resin and polymerized to produce embedding blocks. Ultrathin sections were cut, double-stained, and prepared for observation.

### High-throughput sequencing

Total RNA was extracted from plasma using TRIzol® Reagent (Invitrogen, MA, USA). To remove RNA modifications that could interfere with small RNA library construction, the RNA library, total RNA samples were first pretreated with the following reagents: 3′-aminoacyl (loaded) deacylation to 3′-OH for 3′-adaptor ligation, 3′-cP(2′,3′-cyclic phosphate) removal to 3′-OH for 3′-adaptor ligation, 5′-OH (hydroxyl group) phosphorylation to 5′-P for 5′-adaptor ligation, m1A and m3C demethylation for efficient reverse transcription. The pretreated total RNA samples were then subjected to library preparation using the NEBNext® Multiplex Small RNA Library Prep Set for Illumina® Kit (New England BioLabs, MA, USA). Briefly, RNA was ligated with 3′- and 5′-adapters and cDNA was synthesized, followed by PCR amplification. Finished libraries were sequenced on the Illumina NextSeq 500 system (Illumina, CA, USA) at Aksomics Inc (Shanghai, China) using the NextSeq 500/550 V2 kit (# FC-404-2005, Illumina).

Sequencing quality was checked using FastQC software, and trimmed reads (Pass Illumina quality filter, trimmed 3′-adaptor bases by Cut Adapt) were analyzed using Novo Align software (v2.07.11). Unassigned reads were aligned to other corresponding databases (mRNA/rRNA/snRNA/snoRNA/piRNA/miRNA) alignment. The tsRNA expression levels were measured and normalized to the number of transcripts per million of total aligned tRNA reads (TPM). A paired *P* value < 0.05 was considered statistically significant.

### RNA isolation and RT-qPCR

Total RNA from fresh tissue and cells was isolated using TRIzol (Invitrogen). The expression of selected tsRNA was evaluated by RT-qPCR. The specific primers and probes for the stem-loop RT were purchased from Tsingke; the sequences are listed in Table S1. Relative tsRNA expression was normalized to human U6 levels and analyzed by the comparative threshold method (2^−ΔΔCt^) method analysis. Fold changes were calculated using the 2^−ΔΔCt^ method.

### Statistical analysis

Data are presented as the mean ± standard error of at least three independent assays. GraphPad Prism v. 9.0 was used for statistical analysis. Two-group comparisons were conducted using Student’s t-test. One-way ANOVA was used to compare multiple groups. Statistical significance was set at* P* < 0 0.05.

## Result

### EGCG inhibited the proliferation of NSCLC cells

**Fig. 1:**
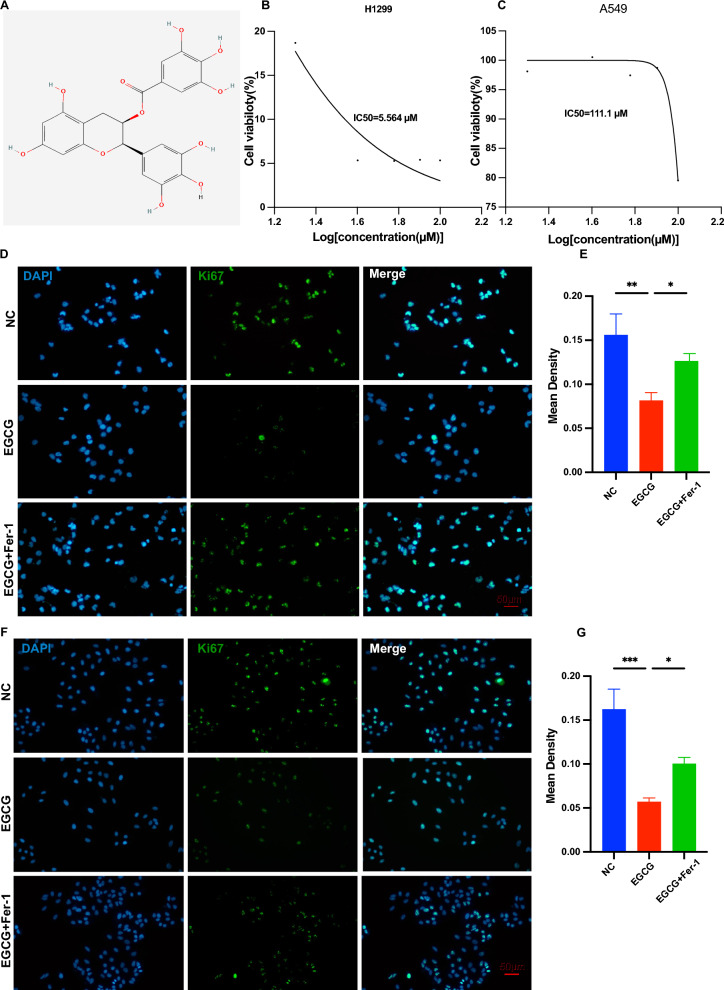
EGCG inhibited the proliferation of NSCLC cells. **A** Illustration of the chemical structure of epigallocatechin gallate (EGCG). **B**, **C** Cell metabolic activity in NSCLC cells was measured by a CCK8 assay after treatment with increasing concentrations of EGCG. The relative IC50 values were determined using non-linear regression analysis with SigmaPlot Software. **D**, **E** Immunofluorescence analysis of Ki-67 expression in H1299 cells treated with EGCG and EGCG + Fer-1, indicating proliferative activity (Scale bar: 50 μm). **F**, **G** A similar analysis of Ki-67 expression in A549 cells under the same treatment conditions showed changes in cellular proliferation (Scale bar: 50 μm). *P < 0.05, **P < 0.01, ***P < 0.001

The molecular structure of EGCG is depicted in Fig. 1A. To evaluate the potential influence of differential EGCG concentrations on the proliferation of H229 and A549 cells, we employed a CCK-8 cell counting assay. As illustrated in Fig. 1B, C, the half-maximal inhibitory concentration (IC50) of EGCG was determined to be 5.564 μM for H1299 cells and 111.1 μM for A549 cells, respectively.

Further, we conducted an immunofluorescence assay, which revealed a decrease in Ki67 expression in both H1299 (Fig. 1D, E) and A549 (Fig. 1F, G) cells, suggesting that EGCG suppressed their proliferation under controlled culture conditions. Interestingly, upon treatment with Fer-1, a well-known ferroptosis inhibitor, Ki67 expression diminished in both H1299 and A549 cells, indicating a possible role of ferroptosis in EGCG-mediated inhibition of cell proliferation.

### EGCG increased levels of Fe^2+^, MDA, ROS, and lipid ROS in NSCLC cells

**Fig. 2:**
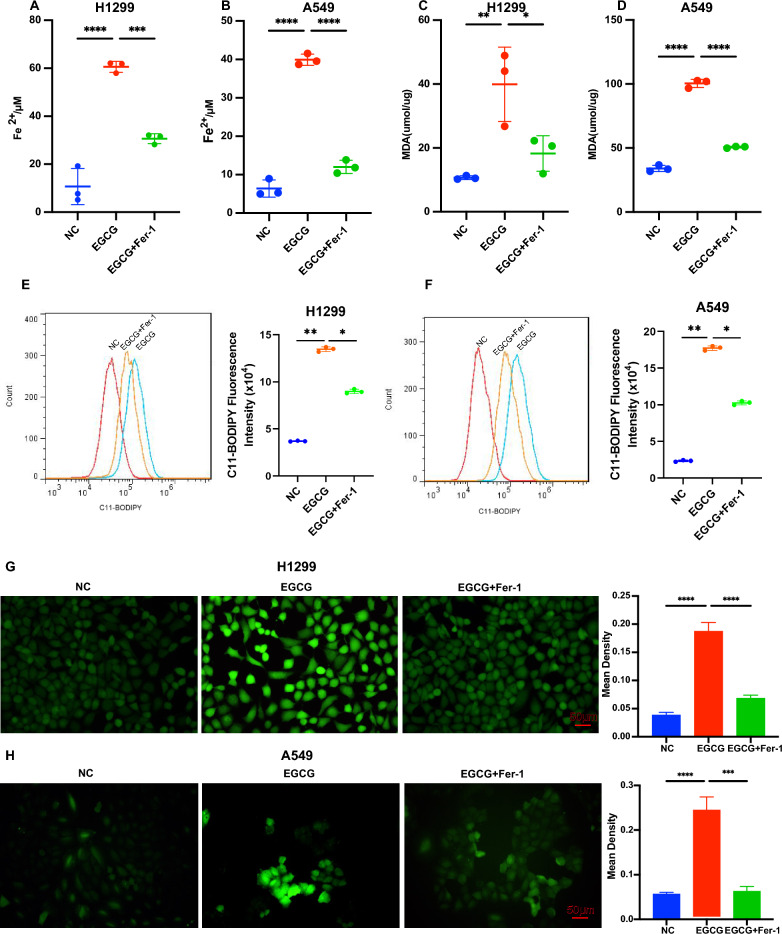
EGCG increased levels of Fe^2+^, MDA, and ROS in NSCLC cells. **A**, **B** Quantification of Fe^2+^ content in NSCLC cells treated with EGCG and EGCG + Fer-1, demonstrating changes in iron metabolism. **C**, **D** Measuring MDA levels in the same cell treatments, assessing lipid peroxidation as an indicator of oxidative stress. **E**, **F** Measurement of the lipid ROS content in NSCLC cells treated with EGCG and EGCG + Fer-1 by flow cytometry. **G**, **H** Detection of intracellular ROS generation using the DCFH-DA probe in NSCLC cells treated with EGCG and EGCG + Fer-1. *P < 0.05, **P < 0.01, ***P < 0.001, ****P < 0.0001

Subsequently, we evaluated the influence of EGCG on Fe^2+^, MDA, and ROS concentrations in NSCLC cells in vitro. Remarkably, compared to the control, EGCG treatment resulted in a significant elevation in Fe^2+^ (Fig. 2A, B) and MDA (Fig. 2C, D) levels in both H1299 and A549 cells, while treating with Fer-1, the levels of Fe^2^ were decreased. Concurrently, lipid ROS levels (Fig. 2E, F) and ROS (Fig. 2G, H) in NSCLC cells were significantly augmented following EGCG treatment and alleviated by Fer-1 treatment.

### EGCG induced ferroptosis in NSCLC cells

**Fig. 3:**
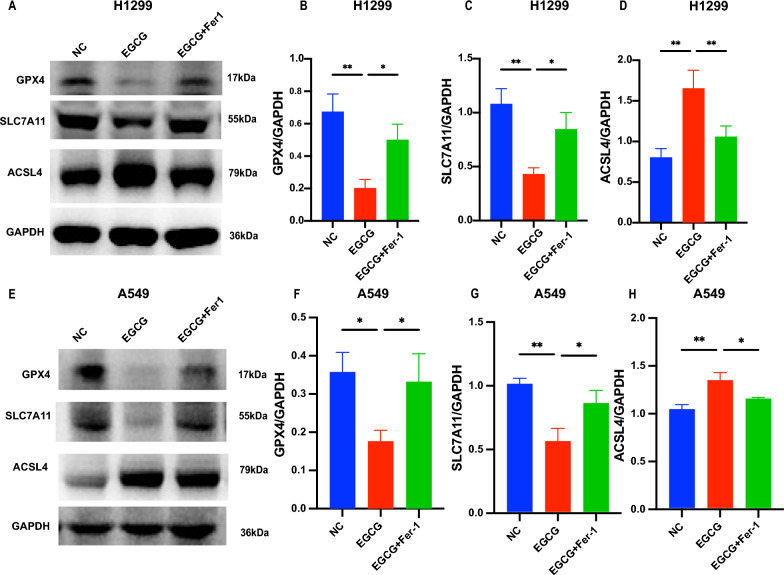
EGCG induced ferroptosis in NSCLC cells. **A**–**D** Western blot analysis shows GPX4, SLC7A11, and ACSL4 expression levels in H1299 cells treated with EGCG and EGCG + Fer-1. **E**–**H** Corresponding Western blot analysis for A549 cells under the same treatment conditions, demonstrating the protein expression of GPX4, SLC7A11, and ACSL4. *P < 0.05, **P < 0.01, ***P < 0.001, ****P < 0.0001

**Fig. 4:**
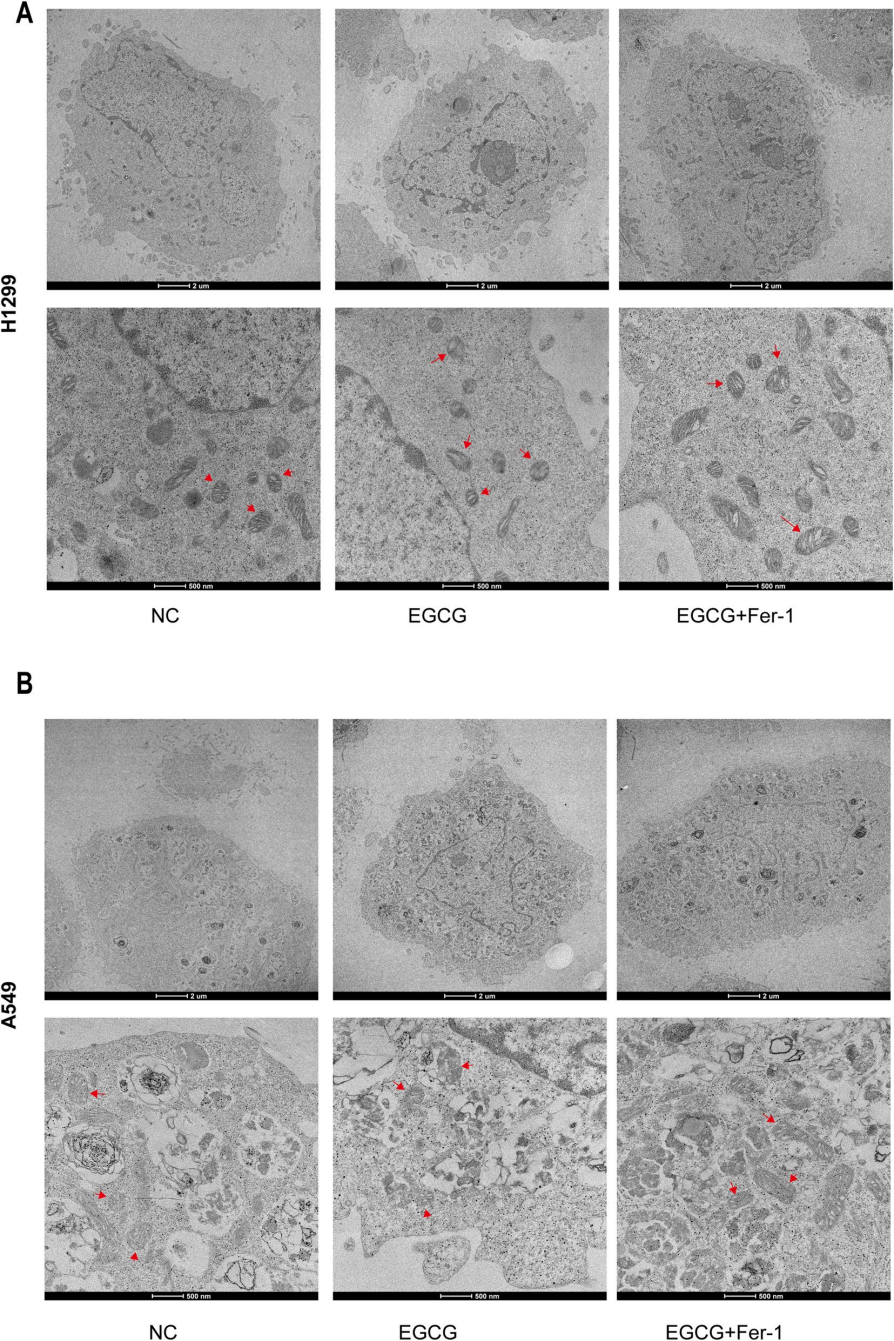
Transmission electron microscope observation of mitochondrial morphology in NSCLC cells. Transmission electron microscopy images of mitochondria in H1299 cells (**A**) and A549 cells (**B**) from control, EGCG-treated, and EGCG + Fer-1-treated groups. The lower panels are partial enlargements of the images above, with red arrows indicating mitochondria

Western blot analyses revealed that in the EGCG-treated group, there was a significant reduction in the protein expression levels of GPX4 and SLC7A11 compared to the control group. Conversely, ACSL4 protein expression was significantly elevated in both H1299 (Fig. 3A–D) and A549 (Fig. 3E, H) cells. Complementing these findings, transmission electron microscopy (TEM) imaging demonstrated that EGCG treatment induced intracellular membrane disintegration and dissolution, mitochondrial swelling and deformation, and abnormal expansion of the endoplasmic reticulum in H1299 (Fig. 4A) and A549 (Fig. 4B) cells. Collectively, these results provide compelling evidence that EGCG can induce ferroptosis in NSCLC cells in vitro*.*

### Overview of tsRNA profiles

**Fig. 5:**
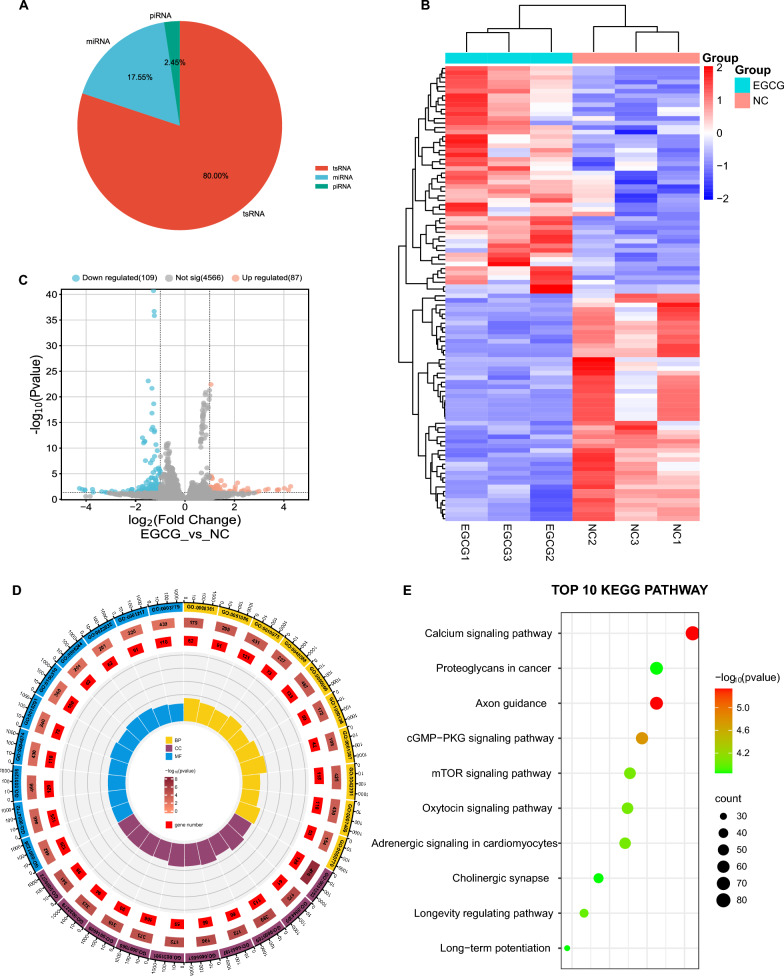
Overview of tsRNAs profiles. **A** Pie chart displaying the distribution of relatively significant smallRNAs identified by high-throughput sequencing. **B** Heatmap of the top 50 significant tsRNAs in A549 cells, scaled and clustered, following treatment with EGCG. **C** Volcano plot analysis illustrating differentially expressed tsRNAs between control (NC) and EGCG-treated groups. Significantly upregulated tsRNAs are marked in red, and downregulated tsRNAs in blue. **D** Gene Ontology (GO) enrichment analysis of the target genes associated with significant tsRNAs, highlighting biological processes, cellular components, and molecular functions. **E** Kyoto Encyclopedia of Genes and Genomes (KEGG) pathway analysis results, show the top 10 enriched pathways influenced by the target genes of significant tsRNAs

Comprehensive small RNA sequencing data unveiled a total of 245 small RNAs that met the criteria of statistical significance (adjusted P < 0.05, and |log2FC| > 1). A breakdown of this cohort revealed 196 tsRNAs, comprising 80% of the total, along with 43 miRNAs (17.55%), and 6 piRNAs (2.45%) (Fig. 5A). The top 50 tsRNAs, scaled for comparison, are depicted in a heatmap (Fig. 5B), whereas a volcano plot provides a visual representation of the tsRNAs (Fig. 5C). Further exploration via Kyoto Encyclopedia of Genes and Genomes (KEGG) and Gene Ontology (GO) enrichment analyses implicated these tsRNAs in a host of biological pathways, including but not restricted to, the Calcium signaling pathway, Proteoglycans in cancer, and Axon guidance (Fig. 5D, E).

### Identify potential tsRNAs

**Fig. 6:**
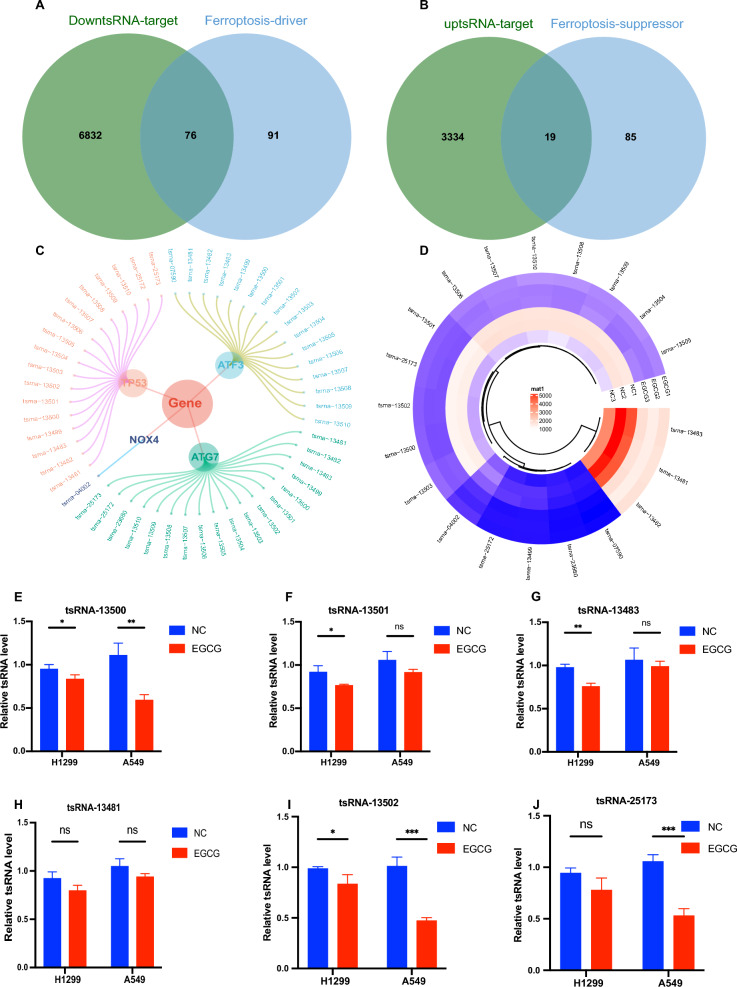
Identify candidate tsRNAs. **A** Venn diagram showing the overlap between genes promoting ferroptosis identified among targets downregulated by tsRNAs. **B** Venn diagram depicting genes that inhibit ferroptosis among targets upregulated by tsRNAs. **C** Classification diagram identifying key genes that promote ferroptosis, including ATF3, ATG7, TP53, and NOX4, which are targeted by 20 downregulated tsRNAs. **D** Circular heatmap illustrating the expression profiles of 20 candidate tsRNAs. (**E**-**J**) RT-qPCR validation of six tsRNAs was selected based on high fold changes and high expression levels. *P < 0.05, **P < 0.01, ***P < 0.001, ns means not significant

To further delineate potential tsRNAs involved in ferroptosis, we conducted an intersection analysis between tsRNAs and ferroptosis-associated target genes. This analysis, visualized in a Venn diagram, identified 76 ferroptosis-promoting target genes amongst the down-regulated tsRNAs (Fig. 6A), and 19 target genes inhibiting ferroptosis amongst the up-regulated tsRNAs (Fig. 6B). We honed in on well-established ferroptosis-promoting genes, specifically ATF3, ATG7, TP53, and NOX4, which were found to be modulated by 20 downregulated tsRNAs (Fig. 6C). A circular heatmap was utilized to display these 20 candidate tsRNAs (Fig. 6D). After considering the expression levels and fold changes of these 20 candidates, we selected 6 tsRNAs for further validation. Subsequent RT-qPCR analysis of these 6 tsRNAs in H1299 and A549 cell lines revealed that two tsRNAs, namely tsRNA-15000 and tsRNA-13502, exhibited significantly reduced expression in the EGCG-treated group compared to the control group (P < 0.05), as illustrated in Fig. 6E–J.

### EGCG promoted ferroptosis in NSCLC by downregulating tsRNA-13502

**Fig. 7:**
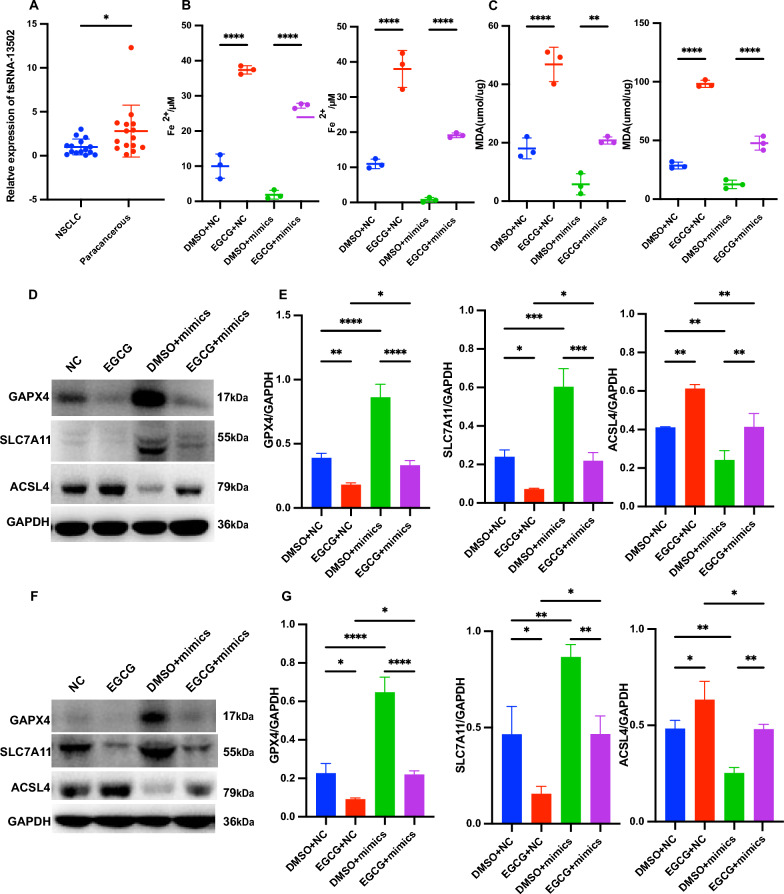
EGCG promoted ferroptosis by downregulating tsRNA-13502. **A** Relative expression of tsRNA-13502 in NSCLC tumor tissues compared to adjacent non-tumor tissues, demonstrating significant downregulation. **B**, **C** Levels of Fe^2+^ and MDA in NSCLC cells treated with DMSO, EGCG, DMSO + tsRNA-13502 mimics, and EGCG + tsRNA-13502 mimics, quantified using commercial assay kits. **D**, **E** Western blot analysis showing expression of GPX4, SLC7A11, and ASCL4 proteins in H1299 cells treated with DMSO, EGCG, DMSO + tsRNA-13502 mimics, and EGCG + tsRNA-13502 mimics. **F**, **G** Similar Western blot analysis was conducted in A549 cells under the same treatment conditions, for GPX4, SLC7A11, and ASCL4 proteins. *P < 0.05, **P < 0.01, ***P < 0.001, ****P < 0.0001

Subsequent investigations entailed a comprehensive evaluation of tsRNA-13502 expression levels in NSCLC and corresponding paracancerous tissues (Fig. 7A). Intriguingly, NSCLC tissues exhibited a significant downregulation of tsRNA-13502 compared to their paracancerous counterparts.

**Fig. 8:**
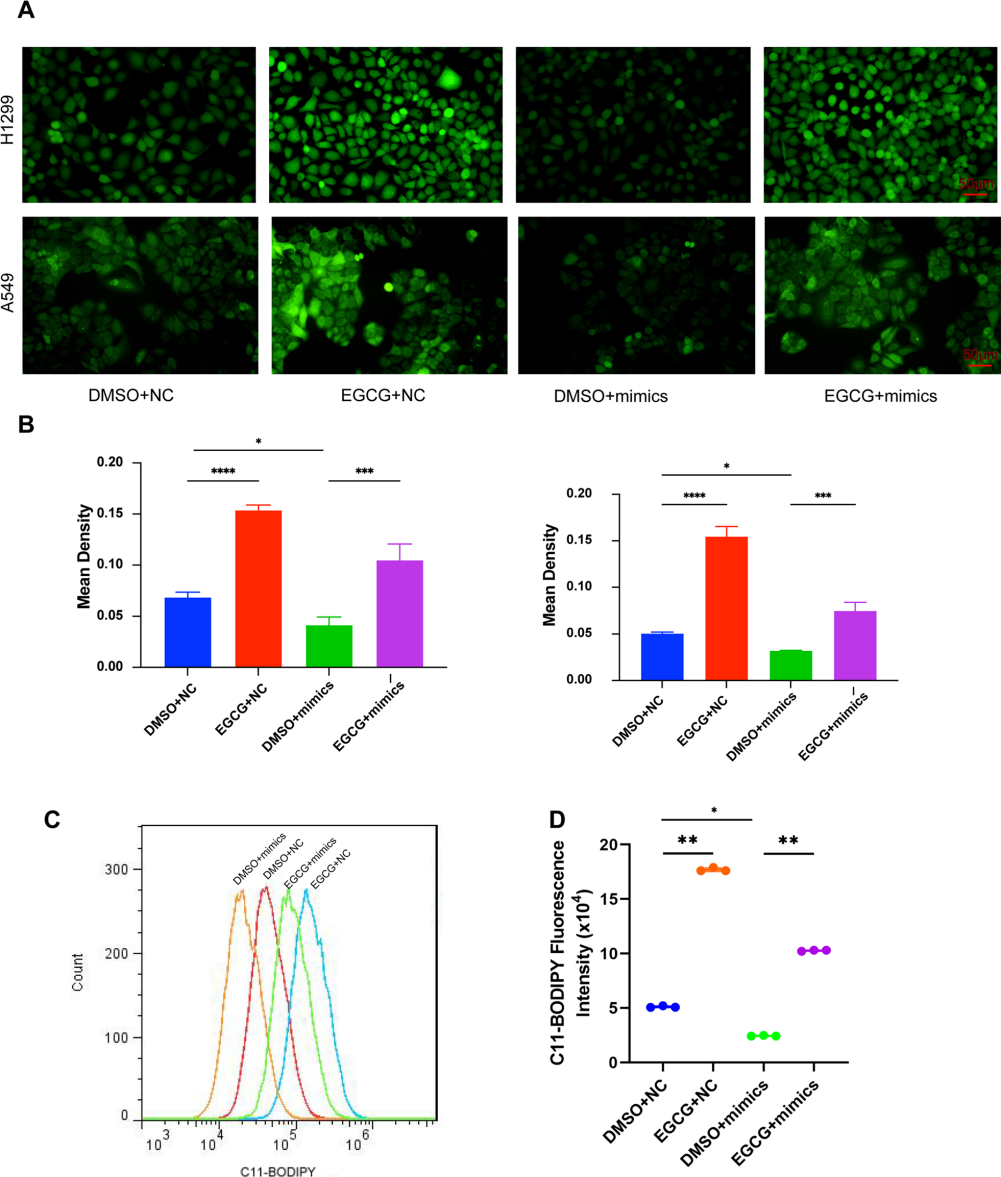
Measure the changes in ROS and lipid ROS levels. **A**, **B** Detection of intracellular ROS using the DCFH-DA probe in NSCLC cells treated with DMSO, EGCG, DMSO + tsRNA-13502 mimics, and EGCG + tsRNA-13502 mimics. **C**, **D** Measurement of the lipid ROS content in NSCLC cells treated with DMSO, EGCG, DMSO + tsRNA-13502 mimics, and EGCG + tsRNA-13502 mimics by flow cytometry. *P < 0.05, **P < 0.01, ***P < 0.001, ****P < 0.0001

To elucidate the functional role of tsRNA-13502, we quantified the concentrations of Fe^2+^ and MDA following varying treatments. Our findings revealed that the EGCG + NC group experienced an augmentation in Fe^2+^ levels relative to the DMSO + NC group, while the EGCG + mimics group demonstrated elevated Fe^2+^ levels in comparison to the mimics + DMSO group (Fig. 7B). A similar trend was observed for MDA levels (Fig. 7C). Further validation through Western blot analyses substantiated that tsRNA-13502 mimetics were capable of enhancing the expression of GPX4 and SLC7A11, whilst concurrently attenuating the expression of ACSL4. This pattern was mitigated in the presence of EGCG (Fig. 7D–G). Additionally, we conducted an exhaustive assessment of ROS and lipid ROS levels across the different groups. The tsRNA-13502 mimics group exhibited the lowest mean ROS fluorescence intensity, whereas the EGCG + mimics group displayed a heightened ROS and fluorescence intensity compared to the mimics + DMSO group (Fig. 8A, B). Flow cytometry indicates that the fluorescence intensity of the EGCG + NC group is higher than that of the DMSO + NC group, and the fluorescence intensity of the EGCG + mimics group is higher than that of the DMSO + mimics group (Fig. 8C, D).

## Discussion

Despite advances in the treatment of non-small cell lung cancer (NSCLC), the mortality rate among patients diagnosed with this disease remains high [1, 5, 25, 26]. Given the challenges in optimally treating recurrent and metastatic disease, the scientific community has made great efforts over the past decade to find new avenues and treatment options for NSCLC patients. EGCG is considered the most abundant compound in green tea and is effective in treating several diseases, including NSCLC [27]. Ferroptosis, a novel form of regulated cell death, has emerged as an attractive therapeutic target in cancer, including NSCLC [28, 29].

Recently, tsRNA has received considerable attention. A growing number of studies have demonstrated that abnormal expression of tsRNA is closely related to disease development [30]. Existing studies have suggested that tsRNA is associated with cancer progression, but the effects of tsRNA on different tumors are heterogeneous [31]. Some tsRNAs were found to be tumor suppressors in breast cancer and chronic [32, 33]. However, studies on tsRNAs in NSCLC are relatively limited. Huang et al. performed sequencing of small RNAs on matched clinical samples consisting of 5 pairs of lung adenocarcinoma tissues and adjacent normal lung tissues. They then validated the expression of three candidate tsRNAs using polymerase chain reaction (PCR) [21].In another study, Gao et al. integrated data from four different databases to identify tsRNA candidates. They performed small RNA sequencing and PCR validation on plasma samples from 50 patients with lung adenocarcinoma and 60 healthy individuals. Their research confirmed the diagnostic value of 11 tsRNAs for lung adenocarcinoma [34, 35].

In the present study, we have uncovered a novel mechanism by which EGCG, a natural compound found in green tea, modulates ferroptosis in NSCLC cells. To elucidate the molecular mechanisms underlying ferroptosis, we intersected the target genes of downregulated tsRNAs with genes known to drive ferroptosis, identifying 76 pro-ferroptotic target genes. Similarly, the intersection of upregulated tsRNA target genes with ferroptosis-suppressing genes yielded 19 genes involved in inhibiting ferroptosis. Among these targets, we focused on well-established pro-ferroptotic genes such as ATF3 [36], ATG7 [37], TP53 [38], and NOX4 [39], which are targeted by 20 downregulated tsRNAs. From these 20 candidate tsRNAs, we selected 6 with the greatest differential expression and highest overall expression levels for further validation. rt-qPCR validation of these 6 tsRNAs revealed that only tsRNA-13500 and tsRNA-13502 showed statistically significant modulation in both H1299 and A549 cell lines. Notably, tsRNA-13502 was more substantially downregulated than tsRNA-13500, leading us to select tsRNA-13502 for further analysis.

Our results highlight the remarkable ability of EGCG to suppress the proliferation of NSCLC cells, which is largely mediated by the induction of ferroptosis. Our results are consistent with previous studies highlighting the proliferation-inhibitory properties of EGCG [40]. However, the mechanistic link to ferroptosis offers new insights. The results of our investigation demonstrated a marked reduction in the expression of tsRNA-13502 in NSCLC cells treated with EGCG, in comparison to control cells. This observation is of particular interest, considering the established role of tsRNAs in a multitude of biological processes, such as the regulation of cell proliferation and cell death. Therefore, the suppression of these tsRNAs by EGCG could potentially be a critical mechanism through which this compound inhibits NSCLC cell proliferation. Corroborating this hypothesis, we observed that NSCLC tissues exhibited a significant downregulation of tsRNA-13502 compared to paracancerous tissues. This observation implies a potential role for this tsRNA in NSCLC progression and suggests it is a potential therapeutic target for the treatment of this disease. Subsequent functional studies unveiled that the overexpression of tsRNA-13502 could counterbalance the EGCG-induced augmentation in Fe^2+^ and MDA levels, both of which are recognized markers of ferroptosis. Furthermore, tsRNA-13502 overexpression could neutralize the EGCG-induced alterations in the expression of key ferroptosis-related proteins, including GPX4, SLC7A11, and ACSL4. These findings suggest that tsRNA-13502 is a key regulator of EGCG-induced ferroptosis in NSCLC cells.

Our investigation adds to the existing literature by offering novel insights into the antiproliferative effects of EGCG on NSCLC cells. Previous studies have noted the anti-cancer effects of EGCG, but the underlying mechanisms have remained largely elusive. Our findings illuminate a previously unexplored pathway involving the induction of ferroptosis and the regulation of tsRNAs, specifically tsRNA-13502, thereby enhancing our understanding of the molecular mechanisms underpinning the anti-cancer effects of EGCG.

Our research, focused on the effects of EGCG on NSCLC via modulation of tsRNA-13502, provides compelling in vitro evidence and insights from clinical samples. However, the lack of animal models in our study restricts our ability to evaluate the full biological and therapeutic implications of these findings in an organismal context. Therefore, while our data suggest promising avenues for treating NSCLC, they must be interpreted cautiously. Future studies incorporating animal models are necessary to confirm these findings and to fully assess the therapeutic potential and safety of targeting tsRNA-13502 with EGCG.

Despite these limitations, our investigation carries significant clinical implications. Given the limited efficacy and considerable side effects of currently available NSCLC treatments, there is a pressing need for the development of novel therapeutic strategies. Our findings suggest that EGCG, a natural compound found in green tea, could potentially be utilized as a therapeutic agent for NSCLC. Moreover, our investigation underscores the potential role of tsRNAs as therapeutic targets in NSCLC. These findings lay the groundwork for the development of innovative therapeutic strategies for NSCLC, potentially improving patient outcomes.

## Conclusions

In conclusion, our investigation illuminates the complex interplay between EGCG, ferroptosis, and NSCLC. The capacity of EGCG to inhibit proliferation through the induction of ferroptosis and its targeting of the ferroptosis-regulating tsRNA-13502 provides a promising basis for future research and the development of innovative therapeutic strategies in NSCLC management.

### Supplementary Information

Supplementary Material 1.

Supplementary Material 2.

## Data Availability

The data supporting this study’s findings are available from the corresponding author, Jie Huang, upon reasonable request.

## Abbreviations

NSCLC: Non-small cell lung cancer
EGCG: Epigallocatechin-3-gallate
RT-qPCR: Real-time quantitative polymerase chain reaction
ROS: Reactive oxygen species
tsRNAs: TRNA-derived small RNAs
tiRNAs: TRNA-derived RNAs
tRFs: TRNA-derived fragments
LUAD: Lung adenocarcinoma
MDA: Malondialdehyde
Fe^2^^+^: Ferrous iron
TEM: Transmission electron microscopy
KEGG: Kyoto Encyclopedia of Genes and Genomes
GO: Gene Ontology
