# Supplementary figures and images for: Epigallocatechin gallate modulates ferroptosis through downregulation of tsRNA-13502 in non-small cell lung cancer
Source: Cancer Cell Int. 2024 Jun 5;24:200. doi: 10.1186/s12935-024-03391-5 (PMC11155022; doi:10.1186/s12935-024-03391-5)

Supplemental Figures


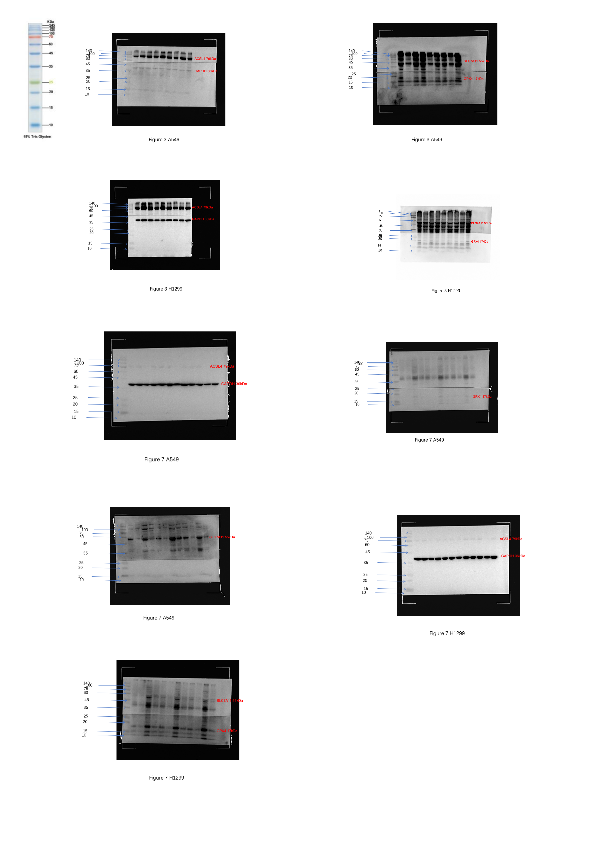


**Figure S1:** **unprocessed original full blot/gel images**

Supplement: Supplementary file 2 — Supplementary Material 2. [file 12935_2024_3391_MOESM2_ESM.docx]
